# Low birth weight among infants and pregnancy outcomes among women living with HIV and HIV-negative women in Rwanda

**DOI:** 10.21203/rs.3.rs-3467879/v1

**Published:** 2023-10-23

**Authors:** Natalia Zotova, Athanase Munyaneza, Gad Murenzi, Gallican Kubwimana, Adebola Adedimeji, Kathryn Anastos, Marcel Yotebieng, CA-IeDEA CA-IeDEA

**Affiliations:** Albert Einstein College of Medicine; Research for Development (RD Rwanda) and Rwanda Military Hospital; Research for Development (RD Rwanda) and Rwanda Military Hospital; Research for Development (RD Rwanda) and Rwanda Military Hospital; Albert Einstein College of Medicine; Albert Einstein College of Medicine; Albert Einstein College of Medicine; Albert Einstein College of Medicine

**Keywords:** Low birth weight, pediatrics, pregnancy outcomes, women living with HIV, Rwanda, Sub-Saharan Africa

## Abstract

**Introduction:**

In utero exposure to HIV and/or triple antiretroviral therapy (ART) have been shown to be associated with preterm births and low birth weight (LBW), but data from low-resources settings with high burden of HIV remain limited. This study utilized retrospective data to describe pregnancy outcomes among Rwandan women living with HIV (WLHIV) and HIV-negative women and to assess the association of HIV and ART with LBW.

**Methods:**

This study used data from a large cohort of WLHIV and HIV-negative women in Rwanda for a cross-sectional analysis. Retrospective data were collected from antenatal care (ANC), delivery, and Prevention of Mother to Child Transmission (PMTCT) registries within the Central Africa International Epidemiology Databases to Evaluate AIDS (CA-IeDEA) in Rwanda. Data from women with documented HIV test results and known pregnancy outcomes were included in the analysis. Analyses for predictors of LBW (< 2,500 g) were restricted to singleton live births. Logistic models were used to identify independent predictors and estimate the odd ratios (OR) and 95% confidence intervals (CI) measuring the strength of their association with LBW.

**Results and discussion:**

Out of 10,608 women with known HIV status and with documented pregnancy outcomes, 9.7% (n = 1,024) were WLHIV. We restricted the sample to 10,483 women who had singleton live births for the analysis of the primary outcome, LBW. Compared with HIV-negative women, WLHIV had higher rates of stillbirth, preterm births, and LBW babies. Multivariable model showed that WLHIV and primigravidae had higher odds of LBW. Lower maternal weight and primigravidae status were associated with greater odds of LBW. Among WLHIV, the use of ART was associated with significantly lower odds of LBW in a bivariate analysis. Even in a sample of relatively healthier uncomplicated pregnancies and women who delivered in low-risk settings, WLHIV still had higher rates of poor pregnancy outcomes and to have LBW infants compared to women without HIV. Lower maternal weight and primigravidae status were independently associated with LBW. Given that supplementary nutrition to malnourished pregnant women is known to decrease the incidence of LBW, providing such supplements to lower-weight WLHIV, especially primigravidae women, might help reduce LBW.

## Introduction

Following the 2010 World Health Organization (WHO) guidelines [1], some countries, for pragmatic reasons, adopted the Option B (triple antiretroviral therapy (ART) for all pregnant women living with HIV (WLHIV) for the prevention of mother-to-child transmission (PMTCT) of HIV starting as soon as possible during pregnancy and continuing through the end of all breastfeeding). In its 2013 updated guidelines the WHO recommended that for PMTCT, lifelong ART be expanded to all pregnant and breastfeeding WLHIV regardless of CD4 count (Option B+) [2]. By 2015, 91% of the 1.1 million women globally receiving ART to prevent mother-to-child transmission were on lifelong therapy [3]. The increased availability of ART for pregnant women has dramatically reduced transmission of HIV from mother to infant [4]. One consequence of this success in PMTCT is that millions of HIV-uninfected infants are being exposed in utero and up to 2 years of age via breastfeeding to both HIV and multiple antiretroviral drugs (ARVs) for which there are limited data on long term safety [5].

Studies prior to the option B/B + era showed that, compared with children born to HIV-negative mothers, children who are HIV-exposed and uninfected (CHEU) have an increased risk of morbidity and mortality [6–12]. Advanced maternal HIV disease during pregnancy has been associated with an increased risk of morbidity and mortality in CHEU [13, 14] in particular thought preterm birth (PTB) risks and associated LBW. The concentration of HIV in the placenta during fetal development was shown to be inversely correlated with birth weight [15]. By reducing morbidity among mothers with HIV [16–18], universal ART in option B/B + has the potential to indirectly reduce the risk of morbidity and mortality among CHEU. On the other hand, exposure to ART may also negatively impact the long-term health of CHEU. Exposure to ARVs in utero increases the risk of prematurity and low birthweight (LBW) [19–23]. Prematurity and LBW are the main contributors to neonatal death [24, 25]. In addition, LBW infants have increased risk of neurodevelopmental impairment (including cerebral palsy) [26–27], impaired lung function and respiratory morbidities [28], and adult onset of diseases such as type II diabetes mellitus, hypertension and cardiovascular disease [29]. From a meta-analysis of forty-three studies in 21 countries, the summary odds ratio of LBW associated with HIV infection in the mother was 1.7 (95% CI: 1.6, 1.8) [30], a measure which has largely remained constant since 1989. However, most of the studies included in the meta-analysis were prior to universal use of ART for pregnant women.

Given the increasing and near universal coverage of ART among pregnant WLHIV and the growing number of CHEU worldwide [31], updated data on pregnancy outcomes including LBW in particular are needed to inform policy and interventions. The PROMISE trial conducted in 7 countries of East and Southern Africa and India compared 3 ART regimes that WLHIV received during pregnancy and found that TDF-based regimens were associated with higher risks of poor pregnancy outcomes and LBW compared to ZDV [32]. Wide implementation of TDF-based regimens in Central Africa calls for an attention to its effects on CHEU. Any health problems that might be associated with HIV or ART exposure in uninfected children could have important public health significance. Yet, no studies have yet assessed the association between *in utero* HIV and/or ART exposure and LBW in settings with a high HIV burden. This study fills this gap and utilize retrospective data to describe pregnancy outcomes among Rwandan WLHIV and HIV-negative women and to assess the association of HIV/ART with LBW among infants born to WLHIV.

## Methods

### Study design and setting

This study was a cross-sectional analysis of retrospective data conducted within the Central Africa International Epidemiology Databases to Evaluate AIDS (CA-IeDEA). CA-IeDEA is part of an international IeDEA research consortium established in 2005 by the U.S. National Institute of Allergy and Infectious Disease (NIAID) to address high priority HIV/AIDS research questions. CA-IeDEA currently includes 22 sites in Burundi, Cameroon, the Democratic Republic of Congo (DRC), the Republic of Congo, and Rwanda. Rwanda, a country in Central Africa with a population of more than 13 million [33] people, has been implementing Option B + since 2012 proving lifelong ART for pregnant and breastfeeding WLHIV. Rwanda has one of the most successful ART programs in the world, with high rates of HIV diagnosis and ART coverage, along with high rates of retention and viral suppression [34]. In 2019, 97% of pregnant WLHIV in Rwanda received ART for PMTCT [35]. Universal ART availability for pregnant WLHIV in Rwanda has contributed to a significant reduction of mother-to-child HIV transmission to less than 2% since implementation of Option B+ [36, 37] resulting in a growing number of CHEU.

### Data collection and population

From February 2018 to September 2021, trained research nurses visited each of the ten health facilities participating in CA-IeDEA with antenatal care, delivery and PMTCT services. With the help of the facility, they accessed antenatal care (ANC), delivery and PMTCT registries and extracted routinely collected data from those registries. Study data were then entered to and managed using REDCap electronic data capture tools hosted at The Ohio State University [38, 39].

Data from antenatal care and delivery registries were manually extracted and linked across the two registries for all women who received care in the facility starting November 2010 (when the country implemented Option B). In addition to ANC and delivery registries, HIV and PMTCT data were obtained from mother-infant pair registries in PMTCT services. In Rwanda’s health care settings, women having potentially complicated or high-risk pregnancies are mostly transferred to district hospitals for ANC services and childbirth. Our sample thus included data from ANC, delivery, and PMTCT registries on generally healthier uncomplicated pregnancies. Data were obtained retrospectively from all CA-IeDEA affiliated health facilities; a consent waiver was obtained for this secondary analysis of existing data. The study was approved by the Rwanda National Ethics Committee, The Ohio State University Institutional Review Board, and Albert Einstein College of Medicine Institutional Review Board.

For the purposes of this cross-sectional analysis, we restricted the sample to women whose HIV status was known at the time of childbirth (who were tested for HIV at admission to maternity clinics or had valid HIV test results in ANC registries) and who had documented pregnancy outcomes, including stillbirth (a baby who dies after 28 weeks of pregnancy, but before or during birth), preterm birth (gestational age < 37 weeks), and birth weight. Doctors or registered nurses used scales in maternity wards to measure birth weight and along with gestational age, recorded them in delivery registries.

### Outcomes of interest and variable definitions

The primary outcomes of interest were poor pregnancy outcomes including stillbirth, preterm delivery or low birth weight (< 2,500 grams). Other variables considered included women’s age (categorized into three groups: ≤24, 25–34, and ≥ 35 years), marital status (married/cohabiting vs. single/divorced/separated/widowed), mother’s weight at the time of delivery (categorized into three groups < 60, 60–64, and ≥ 65 kg), and primigravidae status (no vs. yes). All infants born to a WLHIV were considered to have been exposed to HIV and/or triple ART (mostly TDF + 3TC + NVP or TDF + 3TC + EFC regimens) in-utero. For WLHIV, we included a variable for the use of triple ART during pregnancy (no vs. yes).

### Statistical analyses

Pregnancy outcomes and other variables were summarized using proportions, means or median as appropriate. Chi-square test was used to compare the frequency of poor pregnancy outcomes – stillbirth, preterm births, and LBW - between WLHIV and HIV-negative women. Logistic regression models were used to estimate the odd ratios (ORs) and 95% confidence intervals (95%CI) assessing the strength of the association between LBW and potential predictors including HIV/ART exposure. We fitted models separately for all sample and for WLHIV. Modelling LBW predictors for all sample of women allowed us to assess the effect of HIV and other co-variates. While restricting the model to WLHIV, we aimed to assess the impact of ART use on the risks of LBW. We modeled predictors of LBW separately for births carried to term and preterm births. Preterm infants are more likely to be low weight, so fitting the model separately for term births allowed to assess the effect of LBW predictors with more precision. Variates that were found to be statistically associated (p < 0.2) with LBW in bivariable models were included in the final multivariable model. All statistical analyses were conducted using Stata Version 16.0. [40]

## Results

**Figure 1:**
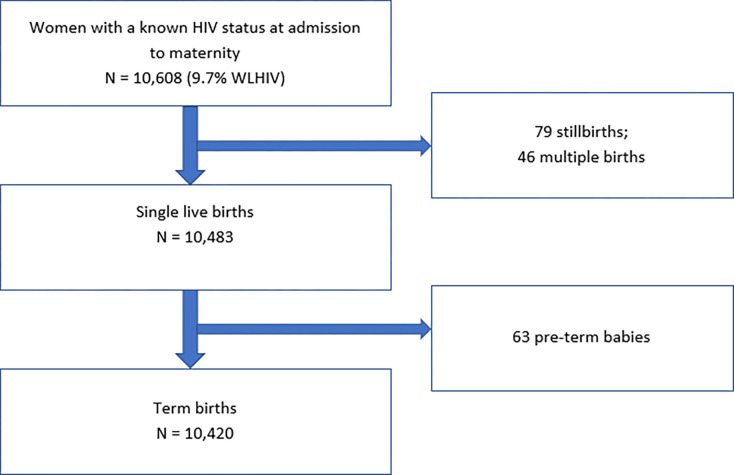
Participants’ flowchart.

Out of 10,608 women with known HIV status and with documented pregnancy outcomes, 9.7% (n = 1,024) were WLHIV (Fig. 1). One-third of the included women (n = 2,679) were aged 24 or less (Table 1). The majority of women (89.1%, n = 6,323) were married or cohabiting and had been pregnant before (non-primigravida) (71.6%, n = 6,654). At the time of delivery, about one-third of women (34.1%, n = 3,486) weighed 60 kilograms or less, 27.4% (n = 2,800) weighed 60–64 kg, and 38.5% (n = 3,939) weighed 65 and more kilograms. Eleven per cent of WLHIV (n = 94) were not using ART at the time of delivery.

### Pregnancy outcomes

Of 10,608 pregnancies, 10,562 (99.6%) were singletons. Of them, 10,483 were single live births and 79 (0.8%) were stillbirths. Of 10,483 single live births, 10,420 (99.2%) were term and 63 (0.8%) were pre-term. The median birth weight was 3,200 grams (IQR 3,000; 3,500) and 363 (3.5%) infants had LBW. WLHIV had higher prevalence of stillbirth (1.2% vs. 0.7%, p = 0.09), preterm babies (1% vs 0.6%, p = 0.09), and LBW babies (4.8% vs 3.3%, p = 0.02), but only the LBW finding was statistically significant.

### Predictors of LBW in the overall sample

In bivariate analyses, compared to HIV-negative women, WLHIV had higher odds of LBW (OR 1.37; 95% CI 0.97, 1.92; p = 0.07) (Table 2). Primigravidae status was also associated with higher odds of LBW (OR 1.98; 95% CI 1.55, 2.53; p < 0.001). Compared to women aged 24 years or less, those 25–34 years (OR 0.61; 95% CI 0.46, 0.81; p = 0.001) and 35 + years (OR 0.66; 95% CI 0.44, 0.99; p = 0.05)] had lower odds of LBW. Odd ratios of LBW were lower among married/cohabiting women (OR 0.54; 95% CI 0.37, 0.78; p = 0.001) compared to divorced/single women and among women who had higher body weight at admission to maternity clinics [60–64 kg vs. 60 or less (OR 0.52; 95% CI 0.39, 0.70; p < 0.001) and 65 + kg vs. 60 or less (OR 0.36; 95% CI 0.27, 0.48; p < 0.001)].

In a multivariable model which included maternal age, marital status, weight, primigravidae status and HIV status, the association between HIV status and LBW was similar (aOR 1.48; 95% CI 0.85, 2.58; p = 0.16), and remained statistically non-significant (Table 2). Higher mother’s weight at delivery and primigravida status remained statistically associated with LBW. When the analysis was expanded to include preterm birth, the association between mother’s HIV status and LBW were statistically significant in bivariate analyses (OR 1.45; 95% CI 1.06, 1.98; p = 0.02), but not significant in a multivariable analysis (aOR 1.55; 95% CI 0.92, 2.61; p = 0.10).

### Predictors of LBW among WLHIV

When the analysis was restricted to WLHIV, in bivariate analyses of term births only primigravidae status and maternal age were statistically associated with LBW, but were not significant in the multivariable analysis (Table 3). When the analysis was expanded to include all singleton births, in bivariate analyses, primigravidae statusmaternal age, and the use of triple ART were found to be statistically associated with LBW. The use of ART was associated with significantly lower odds of LBW (OR 0.41, 95% CI 0.19, 0.89; p = 0.02). Upon adjustment for these co-variates, primigravida status retained a marginally significant association with LBW (aOR 2.36; 95% CI 0.78, 7.17; p = 0.13). The association between ART use and LBW was similar in a multivariable analysis (aOR 0.52, 95% CI 0.18, 1.5; p = 0.02), but was not statistically significant.

## Discussion

In this study, we used data from a large sample of Rwandan women to describe pregnancy outcomes and to investigate predictors of LBW including mother’s HIV status and/or ART use. Globally, the prevalence of preterm birth varies by country and it is estimated to be about 12% in sub-Saharan Africa (SSA) [41]. Overall, with < 1% of preterm birth and stillbirth, the prevalence of poor pregnancy outcomes was relatively low in our sample irrespective of HIV status. Moreover, about 14% of livebirths in SSA are estimated to be LBW [41, 42]. The 3.5% prevalence of LBW in our sample is also lower than the 6.9% reported in the Rwanda recent national demographic health survey [43]. This may be a result of the fact that all 10 participating HIV clinics were in health centers. In the Rwandan pyramidal health system, only uncomplicated pregnancies and deliveries are handled at the level of the health centers. Women with preterm labor, who are more likely to deliver preterm and LBW are referred to the district hospitals.

Consistent with a recent systematic review [44], WLHIV in our sample have higher prevalence of stillbirths, preterm and LBW infants and mothers with higher body weight and multigravidae status have lower odds of delivering a LBW infant. In a meta-analysis published prior to May 2015, Xia et al [30] found that maternal HIV infection was significantly associated with both LBW and preterm delivery. However, they also found that ART did not significantly change the associations of maternal HIV exposure with LBW and preterm delivery. Although our odds ratio of 1.48 measuring the size of the association of HIV/ART with LBW is slightly lower than the 1.73, reported by Xia et al, its 95% CI overlaps their pooled estimate. The majority of WLHIV (89%) were using ART, but we found that the use of ART reduced the odds of LBW by almost a half. The bivariate association between ART use and LBW was only significant in a sample of all singleton live births, yet this suggests the need to promote ART adherence among pregnant WLHIV. Our findings are consistent with a recent registry study from Malawi. Chamanga et al (45) compared adverse birth outcomes among WLHIV and HIV-uninfected women delivering in high (a referral hospital) and low risk (primary healthcare facilities) settings. They showed that rates of LBW and PTB are significantly higher among WLHIV compared to HIV-uninfected and those differences are more pronounced in high-risk settings than in low-risk PHC facilities. This aligns with this study’s findings that showed that even in a sample of relatively healthier uncomplicated pregnancies delivered in low-risk settings, WLHIV still had higher rates of poor pregnancy outcomes compared to women without HIV.

Among WLHIV, ART use was significantly inversely associated with LBW in bivariable analyses and adjustment for age and primigravidae status did not change the estimate substantially. In the PROMISE study [32], women receiving triple ART, especially TDF-based regimens, had significantly higher rate of LBW that those on zidovudine alone. Our findings of a potentially protective effect of ART might be due to uncontrolled/residual confounding as the reasons why some women were not on ART at the time of delivery despite the indication in ANC registries.

It is well known that maternal nutrition during pregnancy has a pivotal role in the regulation of placental-fetal development, that suboptimal maternal nutrition yields LBW [46–48] and nutritional interventions during pregnancy have been shown to positively affect LBW [49, 50]. Thus, the strong association between maternal underweight and LBW that we observed could serve as evidence to support such interventions particularly for WLHIV and primigravidae women.

Our study has some limitations. As discussed above, our sample may be biased towards healthier pregnancies and thus better pregnancy outcomes due to the systematic referral of any potentially complicated pregnancy/delivery to district hospitals. This in turn may explain the relatively low prevalence of poor pregnancy outcomes in our population despite the large number of women and births. Retrospectively linking datasets from ANC and delivery registries allowed for rich data from a large cohort of women. However, data missingness was high for some key variables and guided our decision to not include key important variables like women’s education, occupation and income. These factors could have affected the socioeconomic and nutritional statuses of women during pregnancy.

Despite these limitations, this study has several strengths. It uses data from a large cohort of women in Rwanda, a country that has implemented Option B + providing lifelong ART to all pregnant WLHIV since 2012 and thus allowing us to revisit the association between exposure to HIV (and ART) during pregnancy and pregnancy outcomes in this era of universal ART. To our knowledge, there have been no studies assessing the association between *in utero* HIV and/or ART exposure and LBW in Rwanda and only a few similar studies in other sub-Saharan countries [32, 51, 52]. This study fills a gap in the literature and advances understanding of the effects of HIV and/or ART exposure during pregnancy on birth outcomes, and birth weight, which have important implications for infants’ growth, development, morbidity and mortality in the long-term perspective.

## Conclusions

Even in this era of universal ART during pregnancy, Rwandan WLHIV remain more likely to have LBW infants. Lower maternal weight and primigravidae status were independently associated with LBW. Given that supplementary nutrition to malnourished pregnant women is known to decrease the incidence of LBW, providing such supplements to lower-weight WLHIV, especially primigravidae women, might help reduce LBW.

## Figures and Tables

**Table 1:** Socio-demographic and clinical characteristics of 10,608 women, who had birth outcomes available.

	N (%)*
Age	
<=24	2,679 (32.8)
24–34	4,247 (52)
35+	1,235 (15.2)
Marital status	
Divorced/separated/widowed/never married	771 (10.9)
Married/cohabiting	6,323 (89.1)
Weight at admission, kg	
<60	3,486 (34.1)
60–64	2,800 (27.4)
65+	3,939 (38.5)
Primigravida	
No	6,654 (71.6)
Yes	2,641 (28.4)
ART use (WLHIV only)	
No	94 (11)
Yes	759 (89)

* Frequencies might not add up to the total for the category because of missing data.

**Table 2:** Bivariate and multivariable associations between women’s HIV status, socio-demographic and clinical characteristics, and low birth weight.

	Single live term only					All single live births				
	N* (10,420)	OR (95% CI)	p-value	aOR (95% CI)	p-value	N* (10,483)	OR (95% CI)	p-value	aOR (95% CI)	p-value
HIV status										
Negative	9,473					9,474				
Positive	1,006	1.37 (0.97, 1.92)	0.07	1.48 (0.85, 2.58)	0.16	1,009	1.45 (1.06, 1.98)	0.02	1.55 (0.92, 2.61)	0.10
Age										
<=24	2,635					2,655				
25–34	4,177	0.61 (0.46, 0.81)	0.001	1.02 (0.69, 1.53)	0.91	4,203	0.63 (0.48, 0.81)	< 0.001	0.99 (0.68, 1.44)	0.96
35+	1,207	0.66 (0.44, 0.99)	0.05	1.0x (0.55, 1.86)	0.98	1,213	0.61 (0.41, 0.9)	0.01	0.85 (0.47, 1.54)	0.58
Marital status										
Divorced/separated/widowed/never married	758					761				
Married/cohabiting	6,240	0.54 (0.37, 0.78)	0.001	0.84 (0.52, 1.35)	0.47	6,268	0.59 (0.41, 0.84)	0.004	0.94 (0.59, 1.51)	0.81
Weight at admission, kg										
<60	3,415					3,440				
60–64	2,749	0.52 (0.39, 0.7)	< 0.001	0.48 (0.31, 0.74)	0.001	2,768	0.56 (0.43, 0.73)	< 0.001	0.54 (0.37, 0.81)	0.003
65+	3,881	0.36 (0.27, 0.48)	< 0.001	0.46 (0.31, 0.68)	< 0.001	3,898	0.38 (0.29, 0.5)	< 0.001	0.44 (0.3, 0.65)	< 0.001
Primigravida										
No	6,547					6,577				
Yes	2,588	1.98 (1.55, 2.53)	< 0.001	2.17 (1.46, 3.23)	< 0.001	2,609	2.03 (1.61, 2.55)	< 0.001	2 (1.38, 2.92)	< 0.001

* Frequencies might not add up to the total for the category because of missing data;

** Co-variates that had p-value < 0.2 in bivariate analyses, were included into a multivariable model;

*** OR: odds ratio; aOR: adjusted odds ratio.

**Table 3:** Bivariate and multivariable associations between socio-demographic and clinical characteristics of WLHIV, and low birth weight.

	Single live term births only					All single live births				
	N* (999)	OR (95% CI)	p-value	aOR (95% CI)	p-value	N* (1,009)	OR (95% CI)	p-value	aOR (95% CI)	p-value
Age										
<=24	169					170				
25–34	453	0.56 (0.26, 1.22)	0.15	0.57 (0.22, 1.51)	0.26	459	0.58 (0.28, 1.19)	0.14	0.73 (0.25, 2.15)	0.56
35+	184	0.16 (0.03, 0.72)	0.02	1.0x	(empty)	185	0.20 (0.06, 0.71)	0.01	0.16 (0.02, 1.48)	0.11
Marital status										
Divorced/separated/widowed/never married	64					64				
Married/cohabiting	454	0.84 (0.24, 2.93)	0.78			457	0.93 (0.28, 3.36)	0.97		
Weight at admission, kg										
<60	358					364				
60–64	256	0.61 (0.26, 1.42)	0.25			258	0.63 (0.29, 1.35)	0.23		
65+	353	0.66 (0.32, 1.4)	0.28			355	0.68 (0.35, 1.33)	0.26		
Primigravida										
No	593					598				
Yes	108	2.75 (1.21, 6.24)	0.02	1.78 (0.65, 4.9)	0.26	109	2.43 (1.13, 5.23)	0.02	2.36 (0.78, 7.17)	0.13
Triple ART use										
No	88					92				
Yes	745	0.62 (0.23, 1.66)	0.35			753	0.41 (0.19, 0.89)	0.02	0.52 (0.18, 1.5)	0.23

* Frequencies might not add up to the total for the category because of missing data.

** Co-variates that had p-value < 0.2 in bivariate analyses, were included into a multivariable model;

*** OR: odds ratio; aOR: adjusted odds ratio.

## Data Availability

The datasets analyzed for this study are available upon request to the Executive Committee of IeDEA.
